# Improved adaptation to physical stress in mice overexpressing SUR2A is associated with changes in the pattern of Q-T interval

**DOI:** 10.1007/s00424-020-02401-5

**Published:** 2020-05-27

**Authors:** Rajni Sudhir, Qingyou Du, Andriy Sukhodub, Sofija Jovanović, Aleksandar Jovanović

**Affiliations:** 1 Division of Molecular and Clinical Medicine, Medical School, University of Dundee, Dundee, UK; 2 Department of Basic and Clinical Sciences, University of Nicosia Medical School, Nicosia, Cyprus; 3 Center for Neuroscience and Integrative Brain Research (CENIBRE), University of Nicosia Medical School, Nicosia, Cyprus

**Keywords:** SUR2A, K_ATP_ channel, Cardioprotection, Physical stress, Heart

## Abstract

The purpose of this study was to determine whether increased expression of SUR2A, a regulatory subunit of sarcolemmal ATP-sensitive K^+^ (K_ATP_) channels, improves adaptation to physical stress and regulates cardiac electrophysiology in physical stress. All experiments have been done on transgenic mice in which SUR2A expression was controlled by cytomegalovirus immediate-early (CMV) promoter (SUR2A) and their littermate wild-type controls (WT). The levels of mRNA in heart tissue were measured by real-time RT-PCR. Electrocardiogram (ECG) was monitored with telemetry. The physical adaptation to stress was elucidated using treadmill. We have found that SUR2A mice express 8.34 ± 0.20 times more myocardial SUR2A mRNA than WT (*n* = 8–18). The tolerated workload on exercise stress test was more than twofold higher in SUR2A than in WT (*n* = 5–7; *P* = 0.01). The pattern of Q-T interval from the beginning of the exercise test until drop point was as follows in the wild type: (1) increase in Q-T interval, (2) decrease in Q-T interval, (3) steady stage with a further decrease in Q-T interval, and (4) a sharp increase in Q-T interval. The pattern of Q-T interval was different in transgenic mice and the following stages have been observed: (1) increase in Q-T interval, (2) decrease in Q-T interval, and (3) prolonged steady-state stage with a slight decrease in Q-T interval. In SUR2A mice, no stage 4 (a sharp increase in Q-T interval) was observed. Based on the obtained results, we conclude that an increase in the expression of SUR2A improves adaptation to physical stress and physical endurance by increasing the number of sarcolemmal K_ATP_ channels and, by virtue of their channel activity, improving Ca^2+^ homeostasis in the heart.

## Introduction

Sarcolemmal ATP-sensitive K^+^ (K_ATP_) channels are heteromultimers composed of, at least, two distinct subunits. The pore-forming inwardly rectifying K^+^ channel core, Kir6.2, is primarily responsible for K^+^ permeance, whereas the regulatory subunit, also known as the sulfonylurea receptor, or SUR2A has been implicated in ligand-dependent channel gating. More recently, it has been suggested that the sarcolemmal K_ATP_ channel protein complex may be composed of more proteins than just Kir6.2 and SUR2A, including enzymes regulating intracellular ATP levels and glycolysis [11]. It has been suggested that SUR2A is a rate-limiting factor in assembling sarcolemmal K_ATP_ channels and that the level of this protein regulates the level of fully assembled K_ATP_ channels [7].

Sarcolemmal K_ATP_ channels have been shown to play a crucial role in ischemic preconditioning and myocardial resistance to ischemia [11, 18, 20]. While ischemic heart disease is the leading cause of morbidity and mortality [17], it does not *per se* explain the preservation of K_ATP_ channels through evolution [7]. We have previously shown that and increase in SUR2A and consequent increase in K_ATP_ channel levels increase physical endurance and prolong lifespan [15, 22] while decreased SUR2A levels decrease cellular resistance to metabolic stress [5]. However, no connection between cardiac electrophysiology and improved adaptation to physical stress in mice overexpressing SUR2A has been made so far. In fact, it is yet unknown whether increased expression of SUR2A and the number of K_ATP_ channels affect cardiac electrophysiology at all.

Sarcolemmal K_ATP_ channels are normally closed under physiological conditions to be open in ischemia [7]. The role that this channel plays is yet not fully understood. Therefore, we have decided to take advantage of the phenotype with increased expression of SUR2A [3, 19] and to test whether increase of SUR2A would have any consequence on cardiac electrophysiology during exercise stress.

## Methods

### SUR2A mice

All experiments have been done on male mice overexpressing SUR2A (SUR2A mice) and their littermate controls (WT). Generation, breeding, and genotyping of these mice have previously been described in detail [3, 19]. All experiments conform to the Home Office Regulations in the UK. The experiments have been done under the authority of Project Licences 60/3152 and 60/3925 and mice were sacrificed, when required, by cervical dislocation constituting a Schedule 1 procedure under UK home office regulations.

### Real-time RT-PCR

Real-time RT-PCR was performed as described previously using the same primers [3]. Briefly, total RNA was extracted from the cardiac ventricular tissue of transgenic and wild-type mice using TRIZOL reagent (Invitrogen, Paisley, UK) according to the manufacturer’s recommendations. Extracted RNA was further purified with RNeasy Mini Kit (Qiagen, Crawley, UK); the specific primers for mouse SUR2A, Kir6.2, Kir6.1, SUR1, and SUR2B and all stages of real-time RT-PCR were applied as described in ref. [3]. To determine relative mRNA expression (normalized to the wild type), we have used glyceraldehyde 3-phosphate dehydrogenase (GAPDH) as a control gene. The relative expression ratio(R) of a gene encoding SUR2A is calculated using equation *R* = (*E*_K_)^ΔCP^_k_^(WT-TG)^/(*E*_R_)^ΔCP^_R_^(WT-TG)^ where *E*_K_ is the real-time PCR efficiency of SUR2A gene transcript, *E*_R_ is the real-time PCR efficiency of a reference gene, ΔCP_K_ is the crossing point deviation of wild-type transgene of SUR2A gene transcript, and ΔCP_R_ is the crossing point deviation of wild-type transgene of a reference gene transcript.

### Telemetry

**Fig. 1:**
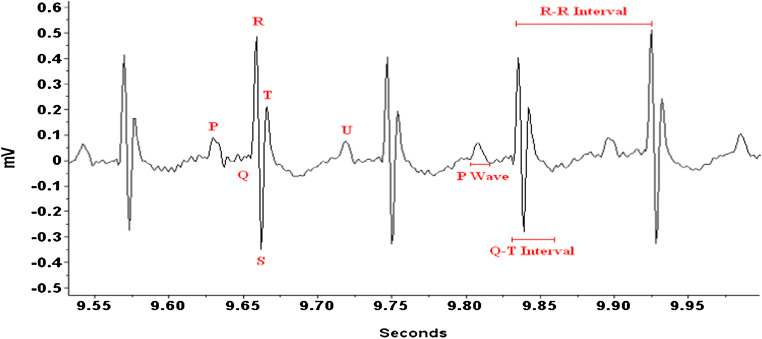
An example of mouse ECG recording and depiction of ECG parameters measured

Telemetry radiotransmitters (ETA-F20, Data Sciences International, St. Paul, MN, USA) were implanted in the peritoneum, and leads were tunneled s.c. in a lead II configuration under isoflurane anesthesia as described in Burgess et al. [1]. Reference points of ECG data were measured as depicted in Fig. 1. Corrected for heart rate, the Q-T interval (*QT*_c_) was calculated by using the Mitchell transformation [*QT*_c_ = *QT*/(*RR*/100)^0.5^], where *RR* (in millisecond) is the interval between previous and current R wave [14].

### Treadmill test

**Fig. 2:**
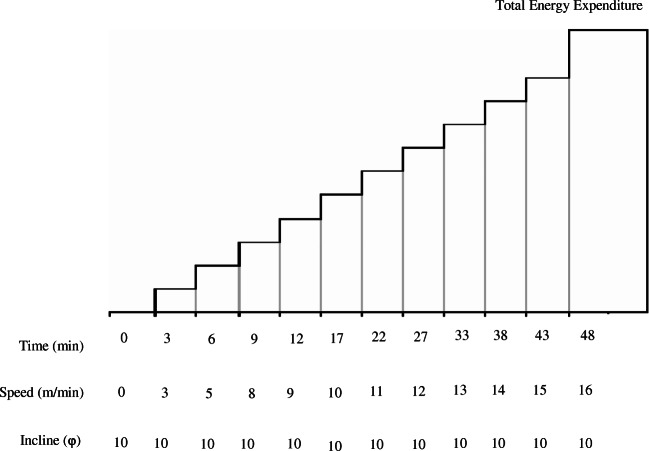
A scheme describing treadmill exercise test

A six-lane treadmill (Columbus Instruments, Columbus, Ohio) was used to perform treadmill tests and determine energy expenditure as described in ref. [19]. The treadmill endurance test consisted of a step-wise increase in velocity over time at a constant incline. The inability to continue with physical activity was determined by the animal being unable to continue test irrespective of encouragement by electric shock (Fig. 2). Energy expenditure was defined as the sum of kinetic (*E*_k_ = m.v^2^/2) and potential energy (*E*p = m.g.v.t.sinφ), where *m* is the animal mass, *v* is the running velocity, *g* is the acceleration due to gravity, *t* is the time elapsed at a given protocol level, and φ is the angle of incline. Energy expenditure was calculated for each time interval during the treadmill endurance test.

### Immunoprecipitation and Western blotting

Sheep anti-Kir6.2 and anti-SUR2 antibodies were used for immunoprecipitation and Western blotting respectively (described in refs. [3, 19]). Sarcolemmal cardiac fraction was obtained as described previously [3] and 40 μg of the epitope-specific Kir6.2 antibody was pre-bound to Protein-G Sepharose beads and used to immunoprecipitate from 50 μg of sarcolemmal fraction protein extract. The pellets of this precipitation were run on SDS polyacrylamide gels for Western analysis. Western blot probing was performed using 1/1000 dilutions of anti-SUR2 antibody and detection was achieved using Protein-G HRP and ECL reagents. The band intensities were analyzed using the QuantiScan software.

### Isolation of single cardiomyocytes

Ventricular cardiomyocytes were dissociated from the mouse using an established enzymatic procedure as we previously described numerous times [3, 19, 21]. In brief, hearts were retrogradely perfused (at 37 °C) with medium 199, followed by Ca^2+^-EGTA-buffered low-Ca^2+^ medium (pCa = 7), and finally low-Ca^2+^ medium containing pronase E (8 mg per 100 ml), proteinase K (1.7 mg per 100 ml), bovine albumin (0.1 g per 100 ml, fraction V), and 200 μM CaCl_2_. Ventricles were cut into fragments in the low-Ca^2+^ medium enriched with 200 μM CaCl_2_ and cells were isolated by stirring the tissue (at 37 °C) in a solution containing pronase E and proteinase K supplemented with collagenase (5 mg per 10 ml).

### Measurement of intracellular Ca^2+^

Intracellular Ca^2+^ in the absence and presence of isoprenaline (500 nM)/glibenclamide (10 μM) was measured using fluo-3 and laser confocal microscopy as previously described [21, 22]. In brief, ventricular cardiomyocytes were loaded with 3.5 μM of fluo-3 acetoxymethyl ester (Fluo-3AM) and transferred to an experimental chamber mounted on the stage of a Zeiss LSM-510 laser-scanning confocal microscope (LSM-510, Zeiss, Göttingen, Germany) filled up with Tyrode’s solution (136.5 mM NaCl, 5.4 mM KCl, 1.8 mM CaCl_2_, 0.53 mM MgCl_2_, 5.5 mM Glucose, and 5.5 mM HEPES-NaOH, pH 7.4) and paced to beat by field stimulation (parameters of the stimulation, 5–20 mV depending on cellular threshold, 5 ms, 0.5 Hz). Intracellular Ca^2+^ was imaged using Ar/UV laser to “excite” the dye at 488 nm. Emission light by photomultiplier tubes was detected at 520 nm. The parameters of image acquisition were similar for all examined cells (mid-cell section thickness was 1 μm and gain was set always at 700 arbitrary units). Images were acquired, viewed, and analyzed using Zeiss Image Examiner Software. Only beating rod-shaped cells with clear striations were used for experimentation.

### Measurement of action potential

Sarcolemmal membrane potential was measured in cardiomyocytes in the absence and presence of isoprenaline (500 nM)/glibenclamide (10 μM) using di-8-ANEPPS and laser confocal microscopy as previously described [3, 21]. Cardiomyocytes were loaded with di-8-ANEPPS according to the manufacturer’s instruction (Invitrogen, Paisley, UK) and the sarcolemma imaged using laser confocal microscopy in line-scan mode (LSM-510, Zeiss, Gottingen, Germany). Cells were stimulated as described in section “Measurement of intracellular Ca^2+^”and scanned under control conditions and then exposed to isoprenaline or isoprenaline/glibenclamide. Fluorescence was detected at 488-nM excitation wavelength and emission was captured at > 505 nM.

### Statistical analysis

Data are presented as mean ± S.E.M, with *n* representing the number of analyzed mice for all in vivo and biochemical experiments and the number of cells for all cellular experiments (no more than 2 cells have been done from the same mouse). Mean values were compared by Student’s *t* test or Mann-Whitney rank sum test where appropriate using SigmaStat program (Jandel Scientific, Chicago, IL). *P* < 0.05 was considered statistically significant.

## Results

### Expression of K_ATP_ channel-forming subunits in wild-type and SUR2A mice

**Fig. 3:**
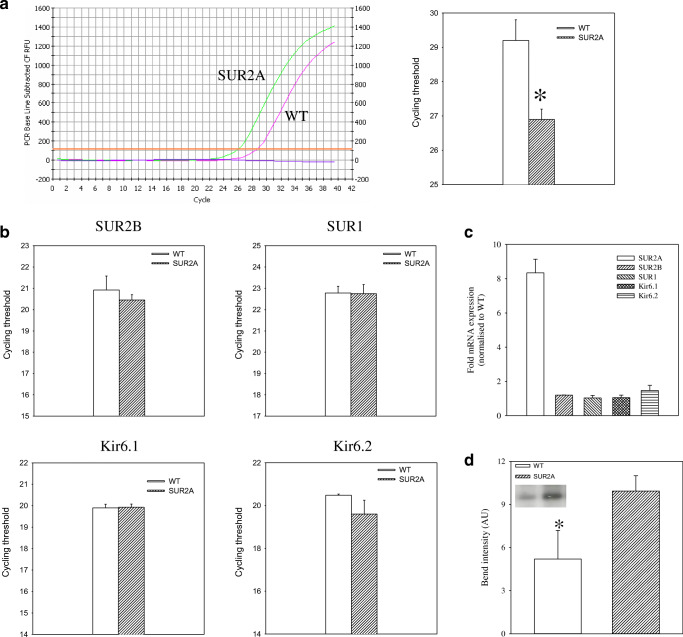
Levels of sarcolemmal K_ATP_ channel subunits in wild-type and SUR2A hearts. **a** Representative progress curves for the real-time PCR amplification of SUR2A cDNA (left panel) from wild-type (WT) and SUR2A (SUR2A) hearts and a corresponding bar graph (right panel) showing cycle threshold for the real-time PCR amplification of SUR2A (*n* = 8–18). *P < 0.01. **b** Bar graphs showing cycle threshold for the real-time PCR amplification of SUR2B, SUR1, Kir6.1, and Kir6.2 from wild-type (WT) and SUR2A (SUR2A) mice. Each bar represents mean ± standard error of the mean (*n* = 8–18). **c** Level of K_ATP_ channel subunits mRNA normalized to the wild type. Each bar represents mean ± SEM (*n* = 8–18). **d** Bar graph depicting bend intensity of Western blotting of anti-Kir6.2 immunoprecipitate from WT and SUR2A sarcolemmal membrane fraction and corresponding original blots in the inset. Each bar represents mean ± SEM (*n* = 3–4) (*P < 0.05)

We have analyzed the levels of SUR2A mRNA in wild-type mice and SUR2A mice. We have confirmed that SUR2A mice had significantly increased mRNA levels of SUR2A (cycling threshold was 29.2 ± 0.6 in wild-type and 26.9 ± 0.3 in transgenics, *n* = 8–18, *P* < 0.01; Fig. 3). No statistically significant difference was observed in mRNA levels of other K_ATP_ channel-forming subunits (Fig. 3). Transgenic mice had 8.34 ± 0.80 times more SUR2A mRNA than the wild type in the heart (Fig. 3). In addition to that, we probed anti-Kir6.2 immunoprecipitate of sarcolemmal fraction with anti-SUR2 antibody, which is a strategy used to measure only those Kir6.2 and SUR2A subunits that physically assemble to form a channel. Western blot has demonstrated that the levels of SUR2 protein were increased in sarcolemma in SUR2A mice when compared with those in WT mice

### Treadmill test in wild-type and SUR2A mice

**Fig. 4:**
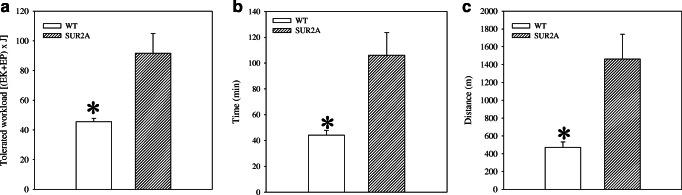
Adaptation to physical stress in wild-type and SUR2A mice. Bar graphs showing energy expenditure (**a**), time spent (**b**), and distance run (**c**) on treadmill of wild-type (WT) and SUR2A (SUR2A) mice. Each bar represents mean ± SEM (*n* = 5–7) (*P < 0.05)

Under the exercise-stress test, SUR2A mice performed at a significantly increased level than the littermate controls (Fig. 4). The tolerated workload, a parameter that incorporates time of effort with speed and incline of the treadmill, was more than twofold higher in transgenic mice (*n* = 7) compared with the wild-type animals (*n* = 5, *P* = 0.01; Fig. 4). On average, transgenic mice were able to run for 1461 ± 279 m on a treadmill (*n* = 7), while wild-type mice have dropped-out after 472 ± 59 m (*n* = 5; Fig. 4). This difference was statistically significant (*P* = 0.01).

### Q-T interval during treadmill test

**Fig. 5:**
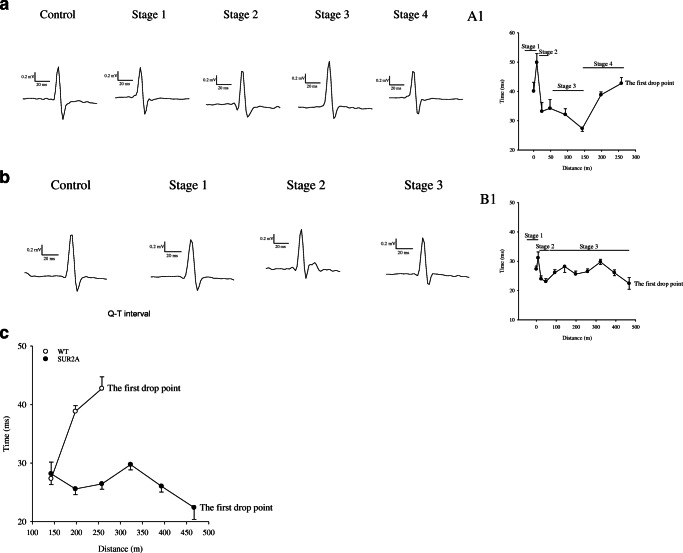
ECG during treadmill stress test. **a** An example of QRST complex changes by treadmill exercise stress test in wild-type mice and corresponding graph describing Q-T interval during the stress test (A1). Each bar represents mean ± SEM (*n* = 4). **b** An example of QRST complex changes during treadmill exercise stress test in SUR2A mice and corresponding graph describing Q-T interval during the stress test (B1). Each bar represents mean ± SEM (*n* = 4). **c** Graph showing Q-T interval in wild-type (WT) and SUR2A (SUR2A) mice in advanced stages of treadmill stress test. Each bar represents mean ± SEM (*n* = 4)

**Fig. 6:**
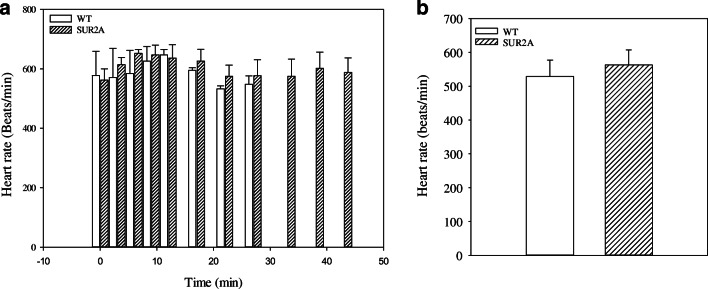
Heart in treadmill test. **a** Heart rate in wild-type (WT) and SUR2A (SUR2A) mice during treadmill test. Each bar represents mean ± SEM (*n* = 4). **b** Heart rate in WT and SUR2A mice 10 min after the treadmill test. Each bar represents mean ± SEM (*n* = 4)

Q-T interval reflects the duration of the action potential [14], while the duration of action potential is shortened by the opening of sarcolemmal K_ATP_ channels [7]. Therefore, we have examined Q-T interval during the treadmill test on 4 WT and 4 SUR2A mice. The pattern of Q-T interval from the beginning of the test until drop point was as follows in the wild type: (1) increase in Q-T interval, (2) decrease in Q-T interval, (3) steady stage with a further decrease in Q-T interval, (4) sharp increase in Q-T interval (Fig. 5). The pattern of Q-T interval was different in transgenic mice and the following stages have been observed: (1) increase in Q-T interval, (2) decrease in Q-T interval, (3) prolonged steady-state stage with a slight decrease in Q-T interval (Fig. 5). In SUR2A mice, no stage 4 (a sharp increase in Q-T interval) was observed. A heart rate just prior to the treadmill test did not differ between and was not different between phenotypes (Fig. 6). In both phenotypes, there was an increase in heart rate in response to treadmill test and there was no statistically significant difference between phenotypes in heart rate during the test (Fig. 6). Heart rate 10 min after the test was not different between the two phenotypes. However, the SUR2A mice that were used for these telemetric measurements spent significantly more time on treadmill than their WT counterparts (2787 ± 306 s for WT and 4387 ± 470 s for SUR2A mice, *n* = 4 for each, *P* = 0.029).

### Action potential and intracellular Ca^2+^ in response to β-adrenoceptor activation

**Fig. 7:**
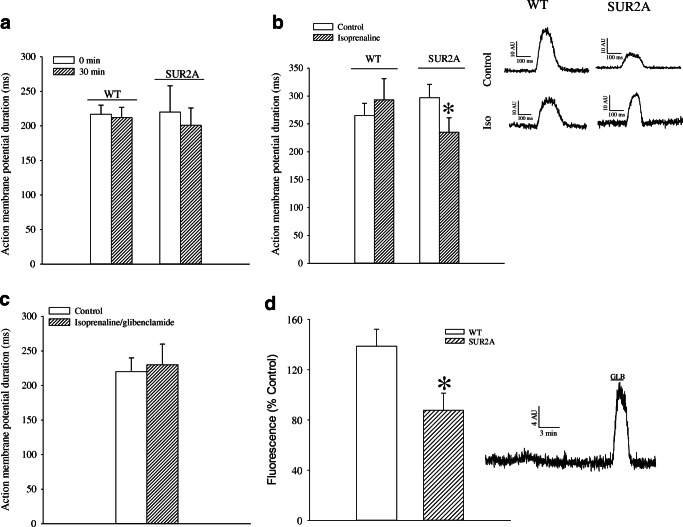
Membrane potential and intracellular Ca^2+^ in cardiomyocytes exposed to isoprenaline. **a, b** Action potential duration in wild-type (WT) and SUR2A (SUR2A) cardiomyocytes in the absence (**a**) and presence (**b**) of 500 nM isoprenaline. Each bar represents mean ± SEM (*n* = 7–14). Inset in **b** depicts original action potential recording corresponding to **b** (*P < 0.05). **c** Action potential duration in SUR2A cardiomyocytes in the absence and presence of 500 nM isoprenaline plus glibenclamide (10 μM). Each bar represents mean ± SEM (*n* = 7–12). **d** Fluo-3 fluorescence in WT and SUR2A cardiomyocytes after 10-min-long exposure to isoprenaline (500 nM). Each bar represents mean ± SEM (*n* = 7–15). Inset in **d** depicts original fluo-3 fluorescence recording of a SUR2A cardiomyocyte exposed to isoprenaline (500 nM) in the absence and presence of glibenclamide (GLB; 10 μM) (*P < 0.05)

If observed differences in ECG were a consequence of different responses of K_ATP_ channels to β-adrenoceptor stimulation, it was logical to expect that evidence of such difference could be observed at the cellular level. Action potential duration did not differ between phenotypes and was similar at point 0 and after 30 min under control conditions (Fig. 7a). When exposed to isoprenaline (500 nM), the action potential was non-significantly prolonged in WT (Fig. 7b). In contrast, action potential was significantly shortened by 500 nM isoprenaline (*P* < 0.001; *n* = 7–14, Fig. 7b) in SUR2A mice. This effect of isoprenaline (500 nM) was blocked by a non-selective K_ATP_ antagonist glibenclamide (10 μM; Fig. 7c). The shortening of action potential in SUR2A mice was associated with prevention of Ca^2+^ loading observed in WT cardiomyocytes (*P* = 0.032, *n* = 7–15; Fig. 7d), which could be reversed by 10 μM glibenclamide (Fig. 7d).

## Discussion

In the present study, we have shown that SUR2A-mediated increase in physical endurance is associated with shortening of cardiac action potential during physical stress.

The general adaptation syndrome is a ubiquitous reaction essential for surviving under conditions of stress such as exertion or fear. It is mediated primarily by a catecholamine surge, and this syndrome generates acute changes in physiological and biochemical functions, which, in turn, improve bodily performance [2]. We have previously demonstrated that non-targeted increased expression of SUR2A generates mice that are characterized by increased physical endurance [19] and that cardiomyocytes with a high level of SUR2A handle Ca^2+^ homeostasis better in response to β-adrenoceptor agonist in comparison with cardiomyocytes containing physiological levels of SUR2A [22]. An increase in SUR2A increases the number of fully assembled K_ATP_ channels [3, 19], which, in turn, leads to faster onset of channel opening in ischemia and increased level of subsarcolemmal ATP [3, 4], both of which foster cardioprotection. In the present study, we confirmed these previous findings and demonstrated that an increased level of SUR2A increases the number of assembled K_ATP_ channels, which, in turn, results in more pronounced K_ATP_ channel activation and improved Ca^2+^ homeostasis under conditions of metabolic stress.

It has been recently suggested that β-adrenergic stimulation of isolated cardiomyocytes from rats subjected to chronic exercise was associated with shortening of action potential due to activation of sarcolemmal K_ATP_ channels [23]. However, the effect of β-adrenoceptor activation on K_ATP_ channels’ activity is complex and might depend on many factors. Few decades ago, it has been described that isoprenaline, a β-receptor agonist, when applied in vitro inhibits pre-activated sarcolemmal K_ATP_ channels [9] and a signaling link between β-adrenoceptors and K_ATP_ channels in non-cardiac tissues has been also described [10]. The regulation of sarcolemmal K_ATP_ channels’ activity is yet to be fully understood as it is controlled by the complex interaction of potentially linked intracellular signaling pathways. In addition to ATP, it has been suggested that the activity of these channels may be regulated by other nucleotides, intracellular pH, lactate, cytoskeleton, protein kinase C, and phosphatidylinositol-4,5-bisphosphate and by the operative condition of the channel itself [7]. An increase in the number of sarcolemmal K_ATP_ channels increases the probability of the channel opening, at least in cells that are metabolically challenged [4, 12, 21]. Indeed, we have observed Q-T interval shortening in SUR2A mice, while Q-T prolongation in WT mice suggesting that increased numbers of sarcolemmal K_ATP_ channels during treadmill exercise resulted in higher probability of opening of these channels, which, in turn, seems to mediate cardiac adaptation to physical stress.

It has been shown that increased expression of SUR2A increases physical endurance over physiological levels [19, 22]. This suggests that the number of sarcolemmal K_ATP_ channels in the heart is an important determinant of cardiovascular response to physical stress. The underlying mechanism of the sarcolemmal K_ATP_ channel–mediated regulation of physical endurance has been proposed to involve the opening of these channels and improvement of Ca^2+^ homeostasis in the heart [22]. When monitoring ECG during the treadmill test, we have noticed a previously unrecognized pattern of ECG changes. A stage-like pattern of Q-T interval would confirm a complexity of the regulation of the cardiovascular system during different degrees of physical stress and would be in accord with the activation of neuronal and hormonal stress systems [8, 13]. It is well established for decades that physical stress is associated with the shortening of Q-T interval [6], but we have here found that there is an initial brief increase in the Q-T interval preceding its shortening. In the wild type, when physical activity has become unsustainable, a sharp increase in Q-T interval was observed, which did not happen in SUR2A mice. An inhibition of an increase in the Q-T interval is consistent with the sarcolemmal K_ATP_ channels counteracting stress-induced prolongation of the action potential during stress as we observed previously at the cellular level [21].

The shortening of the action potential leads to decreased Ca^2+^ influx and prevention of intracellular Ca^2+^ loading, which is very well known to be the main cause of impaired cardiac function during metabolic stress [16]. In cardiomyocytes with physiological levels of SUR2A, there is a slow buildup of intracellular Ca^2+^ when exposed to β-adrenoceptor stimulation [22; present study]. An increased expression of SUR2A prevents Ca^2+^ buildup, showing that upregulation of SUR2A improves Ca^2+^ homeostasis in cardiac cells. This is compatible with the hypothesis that increased number of sarcolemmal K_ATP_ channels protects against intracellular Ca^2+^ loading by shortening ventricular action potentials, which, in turn, improves cardiac response to increased metabolic demands and improves adaptation to physical stress and physical endurance. In the present study, we provided further evidence at the cellular level to support the role of K_ATP_ channels in the regulation of action potential and Ca^2+^ homeostasis and demonstrated that an increase in these channels’ number improves cellular resistance to β-adreneceptor-induced metabolic stress.

In conclusion, this study has shown that increased expression of SUR2A regulates cardiac physiology and improves adaptation to physical stress. The underlying mechanism is likely to involve an increase in the number of sarcolemmal K_ATP_ channels, shortening of action potential, and improved cardiac Ca^2+^ homeostasis.
